# Smoking Status and Cardiometabolic Conditions Among Indigenous Adults in Canada: A Cross-Sectional Study Using the 2022 Canadian Community Health Survey

**DOI:** 10.7759/cureus.109491

**Published:** 2026-05-23

**Authors:** Akinyele Oladimeji, Somto Ojukwu, Yumna W Jandran, Adaeze E Uzozie, Silas Odafe, Marie R Bonhomme

**Affiliations:** 1 Family Medicine, Alberta Health Services, Edmonton, CAN; 2 Internal Medicine, Ebonyi State University Teaching Hospital, Abakaliki, NGA; 3 Internal Medicine, Liaquat University of Medical and Health Sciences, Jamshoro, PAK; 4 General Medicine, University of Nigeria Teaching Hospital, Enugu, NGA; 5 General Internal Medicine, Alberta Health Services, Calgary, CAN; 6 Medicine, State University of Haiti, Faculty of Medicine and Pharmacy, Port-au-Prince, HTI

**Keywords:** canada, cardiovascular disease, cchs, hypertension, indigenous health, smoking status

## Abstract

Background: Cardiometabolic conditions remain a major public health concern among Indigenous populations in Canada. Smoking is a recognized risk factor; however, its independent association with conditions, such as hypertension and cardiovascular disease (CVD), among Indigenous populations remains unclear due to the influence of coexisting cardiometabolic factors, variations in exposure patterns, and the limited availability of population-specific evidence.

Objective: To assess the association between smoking status and cardiometabolic conditions among Indigenous adults in Canada.

Methods: This cross-sectional study used data from the 2022 Canadian Community Health Survey. The analysis included 1,099 Indigenous adults aged 35 years and older, representing a weighted population of 343,048 individuals. Smoking status was categorized as never, former, or current smoker. Outcomes included hypertension and CVD. Survey-weighted logistic regression models with bootstrap variance estimation were used to estimate adjusted odds ratios controlling for demographic and clinical covariates.

Results: Smoking status was not associated with hypertension. Former smokers had an adjusted odds ratio of 1.14 with a 95% confidence interval of 0.51-2.54, and current smokers had an adjusted odds ratio of 1.12 with a 95% confidence interval of 0.45-2.79. Smoking status was also not associated with CVD, with adjusted odds ratios of 0.84 for former smokers and 1.09 for current smokers. Age was strongly associated with hypertension, with individuals aged 50-64 years having an adjusted odds ratio of 2.65 and those aged 65 years and older having an adjusted odds ratio of 4.25. High cholesterol was associated with hypertension at 2.51 and CVD at 4.80.

Conclusion: This study highlights that smoking status was not independently associated with cardiometabolic conditions after adjustment, while age and metabolic factors showed stronger relationships. However, given the cross-sectional design, causal relationships cannot be established, and the findings should be interpreted as associations. These results support integrated risk assessment approaches and highlight the need for further longitudinal research.

## Introduction

Smoking is a leading preventable cause of illness and death globally and is well recognized as a major risk factor for cardiometabolic conditions [1]. One of the most concerning consequences associated with smoking is the increased risk of premature mortality and long-term disability, partly mediated through conditions such as hypertension and cardiovascular disease (CVD) [2,3]. Despite global declines in smoking prevalence in some populations, disparities persist, the rate of smoking continues to be disproportionately high for Indigenous peoples, and according to national (Canada) statistics, they have a higher smoking prevalence than non-Indigenous populations, with both historical and social determinants impacting them [4]. Colonization, socioeconomic inequities, and access to healthcare are all structural factors that have impacted the people of Canada, leaving behind a legacy of smoking behavior that continues today [5].

The most serious public health concerns among Indigenous peoples in Canada are high blood pressure, CVD, and other cardiometabolic conditions [6]. The onset of these conditions can arise from genetic, environmental, and behavioral factors, with smoking being a key modifiable risk factor [7]. Association studies have shown that smoking is associated with increased endothelial dysfunction, arterial stiffness, inflammatory processes, and dyslipidemia, all of which contribute to an increased risk of developing CVD [8]. Furthermore, when combined with other risk factors such as obesity, diabetes, or a lack of physical activity, smoking exerts an additive effect on the burden of disease experienced by Indigenous populations [9,10].

Rich consideration of income inequality, food insecurity, limited availability of culturally appropriate health services, and historical trauma has demonstrated that Indigenous peoples (i.e., First Nations, Inuit, and Métis communities) have an increased frequency of cardiometabolic disease when compared to the total Canadian population [11,12]. The Indigenous people have been affected by living conditions, historical injustices, colonization, land dispossession, and limited access to traditional foods that have impacted nutrition behaviors [13]. These injustices lie in the past and current colonial policies that have contributed to the loss of cultural continuity and jurisdiction of traditional territories [14]. One of the key prevention methods regarding the reduction of risk for cardiometabolic disease is through cessation of smoking [15]. Evidently, cessation at an early age (40 years) has an impressive 90% reduction in the excess risk of death [16].

A substantial amount of research conducted on the relationship of smoking and cardiometabolic disease is focused on the total Canadian population or lacks the ability to disaggregate for the purposes of identifying the health status (disease pattern and/or risk factor) of Indigenous populations [17,18]. Previous research has predominantly focused on singular (e.g., smoking) versus combined (cardiometabolic) health outcomes (e.g., diabetes and heart disease in relation to smoking) and therefore does not provide adequate documentation and identification of the multiple and multifactorial nature of smoking, sociodemographic factors, and health-related variables impacting cardiometabolic health [19].

This study utilizes the Canadian Community Health Survey (CCHS) 2022, which is a cross-sectional survey that has been used to gather data regarding health determinants, health status, and the use and utilization of the health system in Canada [20]. The objective of the study is to evaluate the association between smoking status and cardiometabolic conditions, including hypertension and CVD, among Indigenous adults in Canada. By identifying the patterns of risk linked to smoking, the findings will contribute to the existing literature on culturally appropriate interventions and improved public health strategies.

## Materials and methods

Study design and data source

This study used a cross-sectional design based on data from the 2022 CCHS, a nationally representative survey conducted by Statistics Canada [21]. The survey collects self-reported information on health status, health behaviors, and healthcare use from individuals living in private dwellings across Canada. The survey employs a complex multistage sampling design that incorporates stratification and clustering, and provides person-level sampling weights and bootstrap replicate weights to support population-level inference. The present analysis used the public use microdata file, and all variables were derived according to the survey documentation.

Study population

The study population consisted of Indigenous adults in Canada, identified using the derived Indigenous identity variable that includes First Nations, Métis, and Inuit respondents. The analysis was restricted to individuals aged 35 years and older because the CVD variable was only collected among respondents in this age group, and inclusion of younger individuals would introduce structural missingness due to valid skip responses. Observations with missing data on variables included in the regression models were excluded using a complete case approach. This approach was selected because the proportion of missing data was relatively low-to-moderate across variables and did not suggest systematic patterns related to the primary exposure or outcomes. In addition, the use of multiple imputation was not pursued due to the presence of valid skip responses and the potential for introducing additional assumptions in the imputation process. After applying these criteria, the final analytic sample included 1,099 individuals. The weighted population size represented by this sample was 343,048 Indigenous adults aged 35 years and older in Canada.

Variables and measures

The primary exposure was smoking status, derived from the survey variable that classifies respondents as current, former, or never smokers. Current smokers included both daily and occasional smokers, former smokers included individuals who previously smoked but were not current smokers, and never smokers included lifetime abstainers and experimental smokers, defined in the survey as individuals who have smoked fewer than 100 cigarettes in their lifetime and do not currently smoke. The primary outcomes were hypertension and CVD. Hypertension was defined as a self-reported diagnosis of high blood pressure. This approach may not capture individuals with undiagnosed or unmonitored hypertension, potentially leading to underestimation of true prevalence. CVD was defined as a self-reported history of heart disease, myocardial infarction, or stroke, and was analyzed only among respondents aged 35 years and older in accordance with the survey design. Additional cardiometabolic conditions included self-reported diabetes and high blood cholesterol. Covariates included age group, sex at birth, educational attainment, body mass index category, alcohol consumption pattern, and self-perceived general health. Body mass index was categorized as normal or underweight and overweight or obese based on the survey-derived classification. Alcohol consumption was categorized as non-drinker, occasional drinker, or regular drinker over the past 12 months. Self-perceived health was grouped as excellent or very good, good, or fair or poor. Dietary intake and physical activity variables were evaluated but excluded due to a high proportion of valid skip responses, indicating limited applicability within the survey framework.

Missing data

Missing data were assessed for all variables included in the analysis. The proportion of missing data for individual variables was low to moderate, with the highest levels observed for body mass index (6.03%), smoking status (5.52%), and educational attainment (5.31%). Missingness was lower for diabetes (3.95%) and CVD (3.66%), and minimal for hypertension (0.57%), high blood cholesterol (0.50%), alcohol consumption (0.36%), and self-perceived health (0.14%). A complete case analysis approach was used, whereby observations with missing values for any variable included in the regression models were excluded. This approach was selected because the extent of missing data was limited and the variables with missing values did not show patterns suggesting systematic exclusion related to the primary exposure or outcomes. Valid skip responses were treated as missing for variables where the question was not applicable to certain respondents, including CVD, dietary intake, and physical activity measures. Dietary intake and physical activity variables were excluded from the final analysis due to the high proportion of valid skip responses, which would substantially reduce the analytic sample and introduce potential bias.

Statistical analysis

All analyses accounted for the complex survey design of the CCHS. Person-level sampling weights were applied to produce population-representative estimates, and variance estimation was conducted using bootstrap replicate weights provided with the dataset. Survey design features, including clustering and stratification, were incorporated using survey-specific commands in the statistical software. Descriptive statistics were used to summarize the characteristics of the study population by smoking status. Group comparisons were not formally conducted. Although survey-adjusted cross-tabulations produced Pearson chi-square statistics, these reflect uncorrected test statistics that do not account for the bootstrap variance estimation used in the survey design. As a result, corresponding p-values were not considered appropriate for inference and were not reported. Accordingly, Table 1 presents weighted counts and column proportions without p values or test statistics, consistent with recommended practice when using bootstrap replicate weights. Multivariable logistic regression models were fitted to estimate the association between smoking status and each outcome, including hypertension and CVD, while adjusting for relevant covariates. The covariates included age group, sex, educational attainment, body mass index category, alcohol consumption pattern, diabetes status, high blood cholesterol, and self-perceived general health.

**Table 1:** Characteristics of the study participants by smoking status (n = 1099; N = 343,048) The values are presented as weighted counts and column percentages derived from the Canadian Community Health Survey 2022 [21]. The percentages represent the distribution within each smoking category. Estimates account for the complex survey design using sampling weights and bootstrap variance estimation. Formal group comparisons were not performed because the use of bootstrap replicate weights does not support reliable inference from standard statistical tests in descriptive cross-tabulations. Column percentages may not sum exactly to 100% due to rounding. All the estimates were generated using Stata version 18 [22]. (-) Intentionally left blank, BMI = Body mass index, CVD = Cardiovascular disease

Variable	Never Smoker (N=143,217)	Former Smoker (N=108,531)	Current Smoker (N=91,300)
Sex, n (%)	-	-	-
Male	72,678 (51%)	54,094 (50%)	41,238 (45%)
Female	70,539 (49%)	54,437 (50%)	50,062 (55%)
Age group, n (%)	-	-	-
35-49 years	75,968 (53%)	42,151 (39%)	39,242 (43%)
50-64 years	46,625 (33%)	34,500 (32%)	40,934 (45%)
≥65 years	20,624 (14%)	31,880 (29%)	11,124 (12%)
Education level, n (%)	-	-	-
Less than secondary	6,273 (4%)	8,231 (8%)	19,469 (21%)
Secondary	34,365 (24%)	22,715 (21%)	28,922 (32%)
Post-secondary	102,579 (72%)	77,585 (71%)	42,909 (47%)
BMI classification, n (%)	-	-	-
Normal/Underweight	18,961 (13%)	18,087 (17%)	30,258 (33%)
Overweight/Obese	124,256 (87%)	90,444 (83%)	61,042 (67%)
Alcohol consumption, n (%)	-	-	-
Non-drinker	27,839 (19%)	29,024 (27%)	32,861 (36%)
Occasional	21,290 (15%)	23,437 (22%)	11,541 (13%)
Regular	94,088 (66%)	56,070 (52%)	46,898 (51%)
Diabetes, n (%)	-	-	-
No diabetes	129,335 (90%)	90,004 (83%)	81,999 (90%)
Diabetes	13,882 (10%)	18,527 (17%)	9,301 (10%)
High cholesterol, n (%)	-	-	-
No high cholesterol	119,113 (83%)	81,041 (75%)	73,801 (81%)
High cholesterol	24,104 (17%)	27,490 (25%)	17,499 (19%)
Self-rated health, n (%)	-	-	-
Excellent/Very good	72,068 (50%)	40,927 (38%)	33,479 (37%)
Good	46,332 (32%)	39,852 (37%)	28,747 (31%)
Fair/Poor	24,817 (17%)	27,752 (26%)	29,074 (32%)
Hypertension, n (%)	-	-	-
No hypertension	111,555 (78%)	75,413 (69%)	64,406 (71%)
Hypertension	31,662 (22%)	33,118 (31%)	26,894 (29%)
Cardiovascular disease, n (%)	-	-	-
No CVD	136,933 (96%)	101,429 (93%)	87,126 (95%)
CVD	6,284 (4%)	7,102 (7%)	4,174 (5%)

Adjusted odds ratios with corresponding 95% confidence intervals were reported. All analyses were conducted using Stata version 18 (StataCorp LLC, College Station, TX) [22].

Ethical considerations

This study used publicly available, de-identified data from the CCHS. The dataset does not contain personal identifiers and is accessible for research purposes under Statistics Canada guidelines. As such, this analysis did not require institutional research ethics board approval.

## Results

Table 1 presents the weighted descriptive characteristics of Indigenous adults aged 35 years and older according to smoking status. The results indicate differences in demographic and health characteristics across smoking groups. The proportion of females was higher among current smokers at 50,062 (55%) compared to 70,539 (49%) among never smokers. A greater proportion of current smokers were aged 50-64 years at 40,934 (45%), while individuals aged 65 years and older were more common among former smokers at 31,880 (29%). Educational attainment differed across groups, with post-secondary education reported by 102,579 (72%) of never smokers compared to 42,909 (47%) of current smokers. Overweight or obese status was common in all groups but lower among current smokers at 61,042 (67%) compared to 124,256 (87%) among never smokers. Regular alcohol consumption was reported by 94,088 (66%) of never smokers and 46,898 (51%) of current smokers, while non-drinking was more frequent among current smokers. Diabetes was more common among former smokers at 18,527 (17%) compared to 13,882 (10%) among never smokers. High cholesterol was reported by 27,490 (25%) of former smokers, which was higher than in other groups. Self-rated fair or poor health was more frequent among current smokers at 29,074 (32%) compared to 24,817 (17%) among never smokers. Hypertension was reported by 33,118 (31%) of former smokers and 26,894 (29%) of current smokers, compared to 31,662 (22%) among never smokers. CVD was reported by 7,102 (7%) of former smokers, which was higher than in current smokers at 4,174 (5%) and never smokers at 6,284(4%).

Table 2 presents the adjusted associations between smoking status and hypertension.

**Table 2:** Association between smoking status and hypertension among Indigenous adults (CCHS 2022) Adjusted odds ratios (OR) with 95% confidence intervals (CI) were estimated using survey-weighted logistic regression models accounting for the complex sampling design of the Canadian Community Health Survey (CCHS) 2022 [21], including bootstrap variance estimation. The asterisk (*) indicates significance at the 0.05 level. All the estimates were generated using Stata version 18 [22]. (-) Intentionally left blank

Variable	Adjusted OR	95% CI	p-value
Smoking status	-	-	-
Former smoker vs Never smoker	1.14	0.51-2.54	0.755
Current smoker vs Never smoker	1.12	0.45-2.79	0.814
Age group	-	-	-
50-64 years vs 35-49 years	2.65	1.17-6.04	0.020*
≥65 years vs 35-49 years	4.25	1.65-10.93	0.003*
Sex	-	-	-
Female vs Male	0.90	0.45-1.77	0.753
Education level	-	-	-
Secondary graduate vs Less than secondary	0.83	0.21-3.26	0.793
Post-secondary vs Less than secondary	0.39	0.11-1.42	0.154
BMI classification	-	-	-
Overweight/Obese vs Normal/Underweight	1.97	0.82-4.71	0.129
Alcohol consumption	-	-	-
Occasional drinker vs Non-drinker	1.18	0.48-2.88	0.719
Regular drinker vs Non-drinker	2.06	0.86-4.98	0.107
Diabetes	-	-	-
Diabetes vs No diabetes	1.67	0.65-4.31	0.291
High cholesterol	-	-	-
High cholesterol vs No high cholesterol	2.51	1.21-5.20	0.013*
Self-rated health	-	-	-
Good vs Excellent/Very good	4.32	1.88-9.90	0.001*
Fair/Poor vs Excellent/Very good	3.73	1.46-9.57	0.006*

The results indicate that smoking status was not associated with hypertension. Former smokers had an adjusted odds ratio of 1.14 with a 95% confidence interval of 0.51-2.54, and current smokers had an adjusted odds ratio of 1.12 with a 95% confidence interval of 0.45-2.79. Age showed a strong association with hypertension, with individuals aged 50-64 years having higher odds at 2.65 with a 95% confidence interval of 1.17-6.04, and those aged 65 years and older having higher odds at 4.25 with a 95% confidence interval of 1.65-10.93. High cholesterol was associated with increased odds of hypertension at 2.51, with a 95% confidence interval of 1.21-5.20. Self-rated health also showed strong associations, with individuals reporting good health having higher odds at 4.32 with a 95% confidence interval of 1.88-9.90, and those reporting fair or poor health having higher odds at 3.73 with a 95% confidence interval of 1.46-9.57. Other variables, including sex, education, body mass index, alcohol consumption, and diabetes, were not statistically associated with hypertension.

Table 3 presents the adjusted associations between smoking status and CVD.

**Table 3:** Association between smoking status and cardiovascular disease among Indigenous adults (CCHS 2022) Adjusted odds ratios (OR) with 95% confidence intervals (CI) were estimated using survey-weighted logistic regression models accounting for the complex sampling design of the Canadian Community Health Survey 2022 [21], including bootstrap variance estimation. The asterisk (*) indicates significance at p = 0.05. All the estimates were generated using Stata version 18 [22]. (-) Intentionally left blank

Variable	Adjusted OR	95% CI	p-value
Smoking status	-	-	-
Former smoker vs Never smoker	0.84	0.27-2.60	0.768
Current smoker vs Never smoker	1.09	0.15-7.93	0.932
Age group	-	-	-
50-64 years vs 35-49 years	0.61	0.01-37.21	0.815
≥65 years vs 35-49 years	4.90	0.10-246.59	0.426
Sex	-	-	-
Female vs Male	0.75	0.26-2.17	0.599
Education level	-	-	-
Secondary graduate vs Less than secondary	1.70	0.33-8.70	0.525
Post-secondary vs Less than secondary	1.45	0.32-6.59	0.632
BMI classification	-	-	-
Overweight/Obese vs Normal/Underweight	2.21	0.48-10.17	0.310
Alcohol consumption	-	-	-
Occasional drinker vs Non-drinker	0.37	0.09-1.50	0.163
Regular drinker vs Non-drinker	0.69	0.22-2.12	0.516
Diabetes	-	-	-
Diabetes vs No diabetes	1.97	0.67-5.82	0.218
High cholesterol	-	-	-
High cholesterol vs No high cholesterol	4.80	1.-14.63	0.006*
Self-rated health	-	-	-
Good vs Excellent/Very good	2.15	0.25-18.22	0.483
Fair/Poor vs Excellent/Very good	5.54	0.69-44.31	0.106

**Figure 1:**
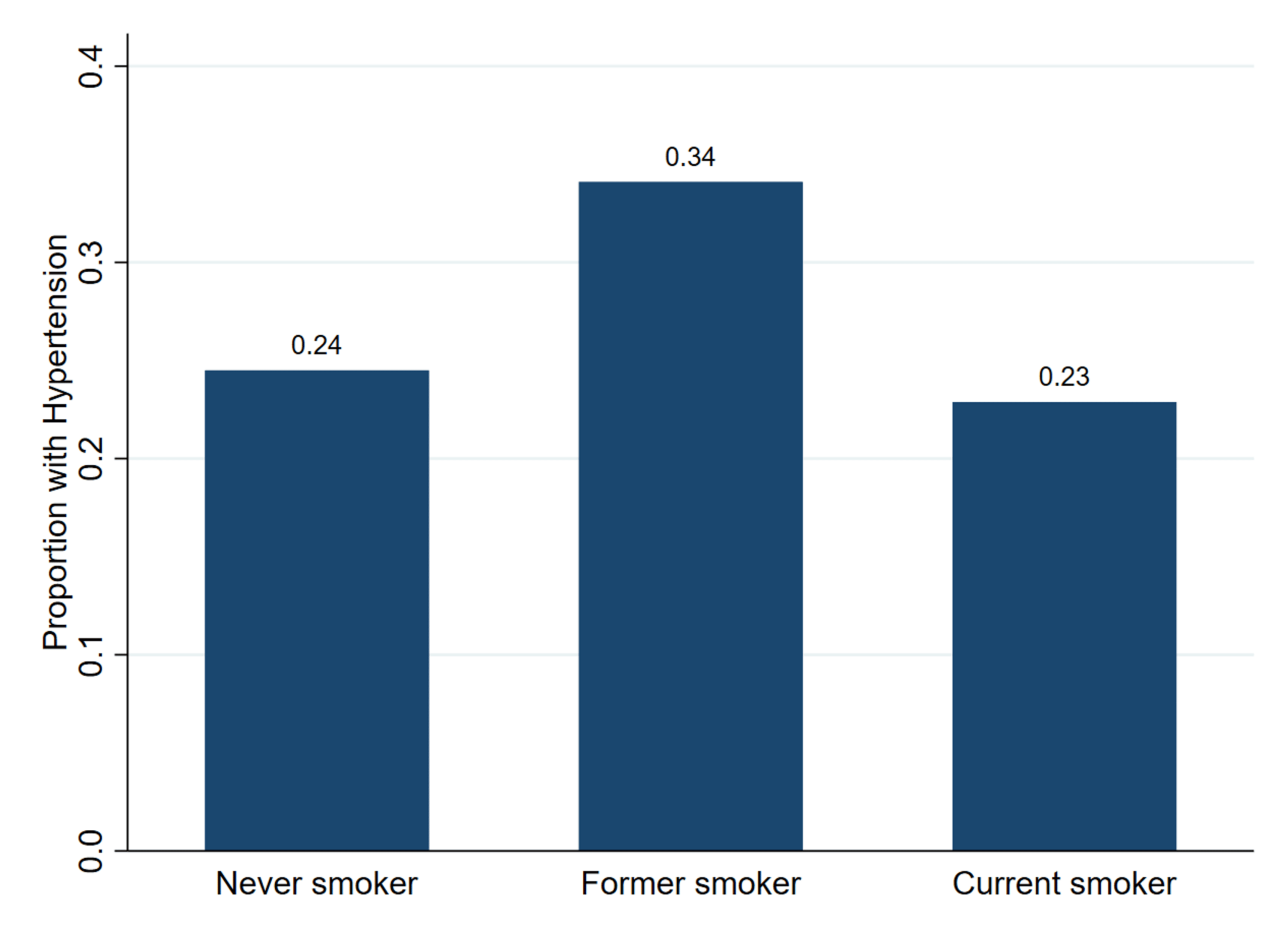
Proportion of hypertension by smoking status

The findings reveal that smoking status was not associated with CVD. Former smokers had an adjusted odds ratio of 0.84, with a 95% confidence interval of 0.27-2.60, and current smokers had an adjusted odds ratio of 1.09, with a 95% confidence interval of 0.15-7.93. High cholesterol was associated with increased odds of CVD at 4.80, with a 95% confidence interval of 1.58-14.63. Other variables, including age, sex, education, body mass index, alcohol consumption, diabetes, and self-rated health, were not statistically associated with CVD, as indicated by wide confidence intervals that included the null value. Figure 1 illustrates the proportion of hypertension across smoking categories.

The figure indicates that the proportion of hypertension was the highest among former smokers at 0.34, followed by current smokers at 0.23, and the lowest among never smokers at 0.24. The pattern suggests a higher burden of hypertension among former smokers compared to other groups.

**Figure 2:**
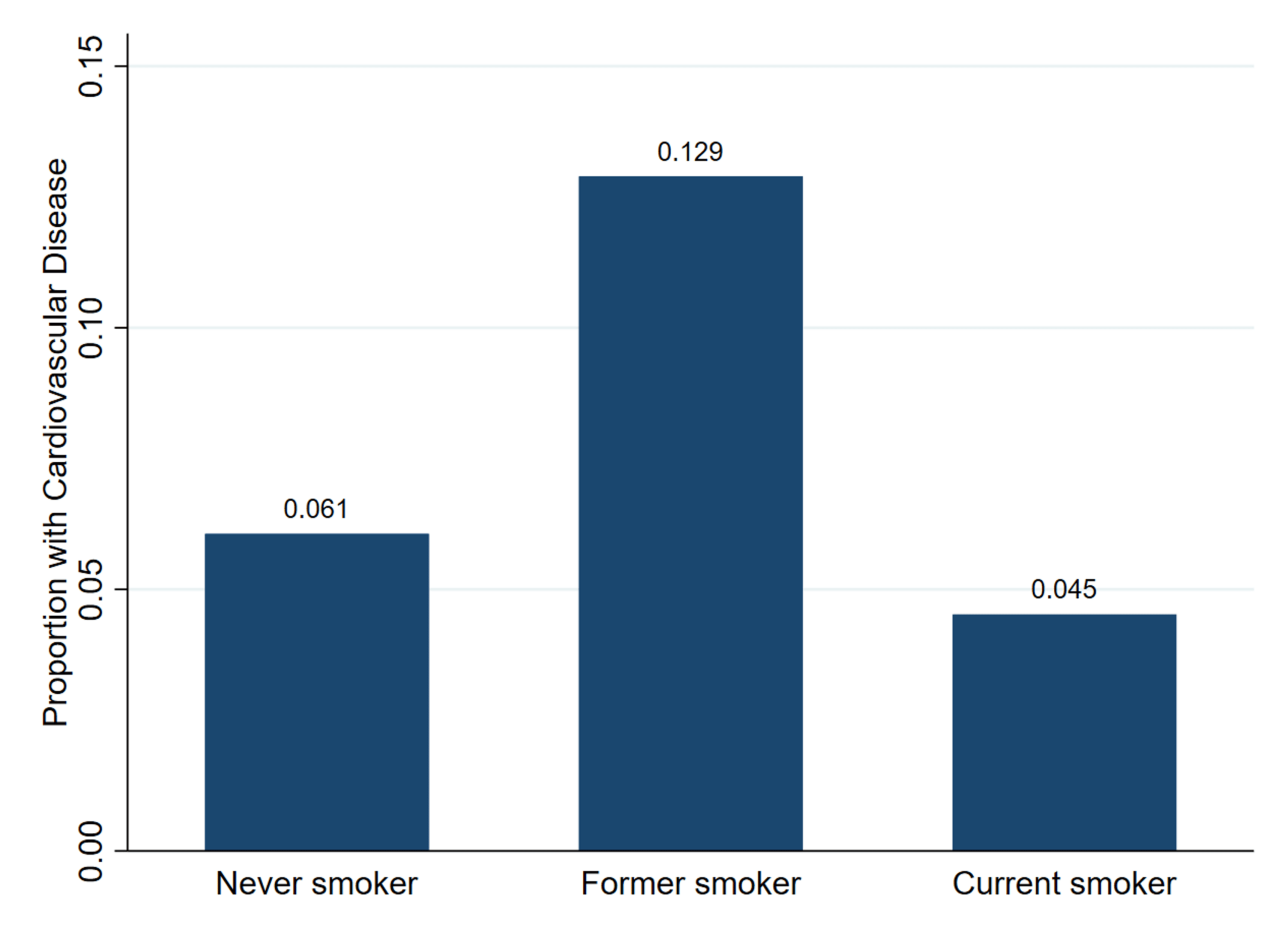
Proportion of cardiovascular disease by smoking status

Figure 2 illustrates the proportion of CVD across smoking categories.

The figure reveals that the proportion of CVD was the highest among former smokers at 0.129, compared to 0.061 among never smokers and 0.045 among current smokers. This pattern shows a higher proportion of CVD among former smokers relative to other smoking categories.

## Discussion

This study examined the association between smoking status and cardiometabolic conditions among Indigenous adults in Canada. The findings indicate that smoking status was not significantly associated with hypertension or CVD after adjustment for demographic and clinical factors. The absence of statistical significance for smoking status in this cross-sectional analysis does not negate its clinical relevance. Smoking remains a key modifiable risk factor for CVD, with extensive evidence documenting its contribution to endothelial dysfunction, inflammation, and accelerated atherosclerosis [3,7,8]. The higher burden of hypertension and CVD observed among former smokers compared to current and never smokers may reflect cumulative tobacco exposure prior to cessation, as well as the possibility that existing illness prompted quitting, a phenomenon noted in prior studies examining smoking patterns and disease risk [15,16]. This "reverse causation" and confounding by comorbidities can obscure associations in cross-sectional data, where temporal relationships and duration of exposure are not captured.

Furthermore, the timing of smoking cessation is critical; cessation at an earlier age has been shown to reduce cardiovascular mortality risk substantially, with a 90% decrease in excess risk when quitting occurs by age 40 [15,16]. The variability in cumulative exposure and cessation timing among participants may have attenuated observed associations in this study. Additionally, Indigenous populations face unique historical and social determinants shaped by colonization, socioeconomic inequities, and limited access to culturally appropriate healthcare, which influence both smoking behaviors and cardiometabolic health outcomes [4,5,11,13]. These complex, multifactorial influences may dilute the independent effect of smoking when analyzed in isolation. In contrast, increasing age, high blood cholesterol, and poorer self-rated health were associated with higher odds of hypertension, while high cholesterol showed a strong association with CVD. Descriptive patterns showed that former smokers had higher proportions of hypertension and CVD compared to never and current smokers. These observations align with prior Canadian evidence showing that cardiometabolic risk is influenced by a combination of age, metabolic conditions, and social determinants rather than a single behavioral factor [1,2,23]. Smoking remains a well-established cardiovascular risk factor across the life course [3,7], yet its effect may vary depending on coexisting conditions and the timing of exposure. The higher burden observed among former smokers may reflect cumulative exposure to tobacco and the presence of existing illness that prompted cessation, which has been noted in previous work examining smoking patterns and disease risk [15,16]. Among Indigenous populations, smoking prevalence and related risk factors are shaped by historical and social conditions, including the legacy of colonization and structural inequities, which influence both exposure and health outcomes [4,5]. Vascular aging is characterized by structural and functional changes in the blood vessels, including increased arterial stiffness and reduced elasticity. These alterations impair the ability of the vasculature to accommodate pulsatile blood flow, thereby elevating systolic blood pressure and myocardial workload, which ultimately predispose individuals to cardiovascular events [8,23]. Several risk factors are implicated in vascular aging, such as arterial hypertension, diabetes mellitus (DM), smoking, and obesity. Interestingly, most of these processes are linked with endothelial dysfunction, the initial step of atherogenesis, which has been proven to be a precursor of adverse cardiovascular outcomes [8]. These contextual factors may contribute to the distribution of cardiometabolic conditions observed in this study.

Current Canadian guidance emphasizes the prevention and management of CVD through risk factor modification, including smoking cessation, healthy diet, and physical activity. National recommendations highlight the importance of early identification and control of hypertension and dyslipidemia, particularly among populations at higher risk [1,2]. Smoking cessation is consistently recommended as a key strategy to reduce cardiovascular risk, with evidence showing reductions in mortality and recurrent events following cessation [15,16]. These recommendations are relevant to the present findings, where high cholesterol and age were strongly associated with adverse outcomes, indicating the need for integrated risk management. Canadian public health approaches also emphasize culturally appropriate care and improved access to preventive services for Indigenous populations, recognizing barriers to care and differences in health system engagement [17]. These principles support targeted interventions that address both clinical risk factors and broader determinants of health.

Several biological and behavioral mechanisms may explain the patterns observed. Tobacco exposure contributes to endothelial dysfunction, inflammation, and vascular injury, which are central processes in the development of CVD [8]. Over time, these effects can interact with other risk factors, such as dyslipidemia and diabetes, leading to progression of disease. The strong association between high cholesterol and both outcomes in this study is consistent with its established role in atherosclerosis. Self-rated health may reflect underlying disease burden, functional status, and psychosocial factors, which have been linked to cardiometabolic risk [12]. In Indigenous populations, additional influences, such as chronic stress, food insecurity, and limited access to health resources, may further shape these pathways [11,13]. Lifestyle factors, including diet and physical activity, are also known to influence cardiometabolic health, although these variables could not be fully examined in the present analysis due to data limitations [9,10].

Strengths and limitations of the study

This study has several strengths and limitations. The use of a large, nationally representative survey with appropriate weighting and bootstrap variance estimation supports population-level inference and enhances the generalizability of findings to Indigenous adults in Canada.

However, several important limitations should be considered. First, the cross-sectional design limits the ability to establish temporal relationships between smoking status and cardiometabolic conditions, and the findings should therefore be interpreted as associations rather than causal effects. In addition, the observed higher burden of cardiometabolic conditions among former smokers may reflect reverse causation, where individuals with existing illness are more likely to quit smoking.

All measures were self-reported, which may introduce reporting and recall bias, particularly for conditions such as hypertension and CVD. This approach may also fail to capture undiagnosed cases, leading to potential misclassification and underestimation of true disease prevalence. Residual confounding remains a concern, as important factors, such as dietary patterns, physical activity, and other behavioral and social determinants, could not be included due to data limitations and may influence both smoking behavior and cardiometabolic risk.

Missing data were handled using complete case analysis, which reduced the analytic sample and may introduce bias if missingness is not completely random. Although the proportion of missing data was relatively low to moderate, the exclusion of observations with incomplete data may affect the precision and representativeness of the estimates. Key variables, such as dietary intake and physical activity, were excluded due to a high proportion of valid skip responses, further limiting the ability to fully account for lifestyle-related confounding.

Additionally, the CVD variable was restricted to individuals aged 35 years and older, which reduced the analytic sample and may limit generalizability to younger populations. The relatively small number of CVD cases is also reflected in the wide confidence intervals observed in regression analyses, indicating limited statistical power to detect associations.

Finally, while the study focuses on Indigenous populations, heterogeneity within these groups may not be fully captured due to the aggregation of First Nations, Inuit, and Métis respondents, which may mask important subgroup differences.

Future research should consider longitudinal designs, incorporate objective clinical measures, and include a broader range of behavioral, clinical, and social determinants to better understand cardiometabolic risk in Indigenous populations.

## Conclusions

This study highlights that smoking status was not independently associated with hypertension or CVD among Indigenous adults after accounting for demographic and clinical factors. Instead, age, high blood cholesterol, and self-perceived health were more strongly related to these outcomes. These findings support a broader approach to cardiometabolic risk that extends beyond smoking alone. The results also point to the importance of integrated prevention strategies that address multiple risk factors within Indigenous populations. Clinical care should continue to prioritize screening and management of modifiable conditions such as dyslipidemia. Future research should use longitudinal data and include a wider range of behavioral and social determinants to better understand patterns of cardiometabolic health in this population.
